# BMI and serum lipid parameters predict increasing risk and aggressive prostate cancer in Chinese people

**DOI:** 10.18632/oncotarget.19790

**Published:** 2017-08-02

**Authors:** Ruizhe Zhao, Gong Cheng, Bing Wang, Chao Qin, Yun Liu, Yongsheng Pan, Jun Wang, Lixin Hua, Weidong Zhu, Zengjun Wang

**Affiliations:** ^1^ Department of Urology, First Affiliated Hospital of Nanjing Medical University, Nanjing 210029, China; ^2^ Department of Urology, Zhongda Hospital Affiliated to Southeast University, Nanjing 210008, China; ^3^ Department of Geratology, First Affiliated Hospital of Nanjing Medical University, Nanjing 210029, China; ^4^ Department of Information, First Affiliated Hospital of Nanjing Medical University, Nanjing 210029, China; ^5^ Department of Urology, School of Medicine, The First People's Hospital Affiliated to Shanghai Jiao Tong University, Shanghai 200080, China

**Keywords:** prostate cancer, obesity, serum lipid parameters, risk, prognosis

## Abstract

**Objectives:**

To determine if obesity and serum lipid parameters are associated with increased risk and more aggressive prostate cancer in Chinese population

**Materials and Methods:**

We conducted a retrospective cohort analysis including 3102 patients. Kruskal-Wallis test for continuous variables and the chi-squared tests for categorical variables were used for univariate comparison of the differences in patient characteristics across BMI categories between different groups. Odds ratios (OR) and 95% confidence intervals (CI) were estimated for the association between prostate cancer and the various patient characteristics. Multivariable Cox proportional hazards regression was performed to assess the risk of prostate cancer recurrence

**Results:**

974 consecutive men were diagnosed as prostate cancer and 700 patients subsequently received radical prostatectomy immediately, and 1031 patients were pathologically diagnosed as biopsy negative. The level of low-density-lipoprotein cholesterol (LDL-c) and total cholesterol was significantly higher and the high-density-lipoprotein cholesterol (HDL-c) level is much lower in prostate cancer patients. Patients with low level of HDL-c, who subsequently received radical prostatectomy, had increased risk of high risk disease. In addition, patients with normal weight were less likely to develop a biochemical recurrence. Combined analysis revealed that obese patients had significantly higher rates of PSA recurrence over time than nonobese patients.

**Conclusions:**

In our study, lipid parameters are supposed to be associated with prostate cancer risk and aggressiveness. Obese men are at increased risk of PSA recurrence after radical prostatectomy.

## INTRODUCTION

Prostate cancer (PCa) is the most prevalent kind of malignancies and the second leading cause of cancer deaths in North American. In Asian, data shows that the incidence of the PCa is much lower compared with the Western countries [1]. However, the rates have risen rapidly in the past two decades in most Asian countries [2], which is even faster than Western world, and in China, PCa has become the most common malignant cancer of male urogenital system. The causes and the development of PCa are not well understood and the reasons for this racial disparity are uncertain. Genetic variants are undoubtedly supposed to be the vital factors. However, study concerning the incidence of PCa between the indigenous American population and Asian immigrants shows that the differences are reducing [2], reflecting that environmental factors, in particular the influence of diet and the ‘‘Western lifestyle,’’ are supposed to contribute to the pathogenesis of this disease.

Obesity, which is usually concerned with high body mass index (BMI), is being increasingly prevalent worldwide and becoming a significant public health concern in China, being linked with diseases including cardiovascular disease, type 2 diabetes and incidence and mortality from a variety of adult cancers like breast cancer [3–5]. Many studies have been conducted to explore the association between obesity and the occurrence of prostate cancer, but data about the effects of obesity on PCa are controversial. Some studies suggested that higher body mass index (BMI) has a positive correlation with PCa risk [6–8] whereas others found no increased risk [9]. Recently, several large studies demonstrated that higher BMI is associated with a lower PCa risk of but a higher risk of high grade PCa [10, 11]. In addition, some studies found that patients underwent radical prostatectomy (RP) with a higher BMI are more likely to have advanced disease [12] and experience biochemical recurrence [13, 14]. PSA has been widely used for early detection of the disease, however obesity doesn’t elevate level of PSA, which means PSA cannot completely reflect the risk of potential risk and advancement of the disease [15, 16]. Although PSA isoforms like were proved to be effective for predicting prostate cancer in obese people [15], they are still not been widely applied in China.

The association between the levels of cholesterol and PCa has been explored for a long time. However, the conclusions are inconsistent. Some studies demonstrated a positive association between cholesterol and prostate cancer mortality [17, 18], while inverse results were found in other investigations [19, 20]. Some reports revealed that patients with high level of serum cholesterol are more likely to have prostate cancer progression or high grade PCa [21–23]. LDL-c, HDL-c and serum triglycerides are also reported to be associated with PCa, but the evidence is limited [24–27].

Growing evidence has demonstrated that obesity and aberrant lipid profile were associated with the PCa risk and outcome of RP in many other races. However, few data on Chinese population was available. In this study, we investigated the association between BMI and lipid profile and the risk of prostate cancer. Furthermore, PCa recurrence risk was also examined by different levels of BMI and lipid profile to demonstrate the risk factors of PCa-specific outcomes after RP.

## RESULTS

Table 1 demonstrated the demographics and disease-specific factors between the BPH and PCa patients. Of the 2005 men included in this study, 974(48.5%) were diagnosed as PCa. In comparison with non-PCa group, PCa group has significant higher level of PSA (6.00±3.03 vs. 73.57±407.07, *P* < 0.001), and more high level of PSA (>20 ng/ml) of observed in PCa patients. In addition, the level of LDL-c and total cholesterol was significantly higher (*P* = 0.001 and *P* = 0.020 respectively) and the HDL-c level is much lower in PCa patients. More PCa patients had a higher LDL-c and total cholesterol level and lower HDL-c level (*P* = 0.005, *P* = 0.015 and *P* = 0.001 respectively). No difference was found in age, hypertension, diabetes and BMI condition between two groups (*P* = 0.119, *P* = 0.078, *P* = 0.322 and *P* = 0.181 respectively).

**Table 1:** Comparison of by demographics and disease-specific factors between biopsy negative people and PCa patients

	non-PCa	PCa	*P*
	N=1031(%)	N=974(%)	
Demographics			
Age at diagnosis	68.83±7.40	68.74±6.43	0.119
<55	59(5.72%)	24(2.46%)	<0.001
55-64	267(25.90%)	199(20.43%)	
65-74	508(49.27%)	572(58.73%)	
75+	197(19.11%)	179(18.38%)	
Obesity (BMI)	28.48±7.01	28.68±6.99	0.078
<25	272(26.38%)	302(31.01%)	0.126
25-30	329(31.91%)	303(31.11%)	
30-35	224(21.73%)	195(20.02%)	
>35	206(19.98%)	174(17.86%)	
Hypertention	336(32.59%)	345(35.42%)	0.181
Diabetes	176(17.07%)	183(18.79%)	0.322
Lipid profile			
HDL-c(mmol/L)	1.26±0.29	1.22±0.31	**0.001**
HDL-c(<1.03 mmol/L)	153(14.84%)	198(20.33%)	**0.001**
LDL-c(mmol/L)	2.96±0.70	3.11±0.72	**<0.001**
LDL-c(>4.1 mmol/L)	63(6.11%)	92(9.45%)	**0.005**
Total cholesterol(mmol/L)	4.86±1.04	4.94±0.97	**0.02**
Total cholesterol(>6.2 mmol/L)	72(6.98%)	98(10.06%)	**0.015**
Total Triglyceride(mmol/L)	1.57±1.13	1.51±1.73	0.08
Total Triglyceride(>2.25 mmol/L)	117(11.35%)	87(8.93%)	0.053
Disease characteristics			
Diagnosis of PSA(ng/ml)	6.00±3.03	73.57±407.07	**<0.001**
<4	73(7.08%)	32(3.29%)	**<0.001**
4–9.9	890(86.32%)	245(25.15%)	
10–19.9	54(5.24%)	279(28.64%)	
20+	14(1.36%)	418(42.92%)	
Pathological stage			
T1–T2a		262(26.90%)	
T2b–T2c		467(47.95%)	
T3+		245(25.15%)	
Gleason score			
≤6		326(33.47%)	
≥7		648(66.53%)	
Capsular penetration		82(8.42%)	
Seminal vesicle invasion		103(10.57%)	
Lymph node involvement		117(12.01%)	

According D'amico classification, PCa patients were categorized into three risk groups to evaluate the risk of recurrence. Low risk refers to PSA less than or equal to 10 ng/ml, Gleason score less than or equal to 6, or clinical stage T1-2a. Intermediate risk refers to PSA between 10 and 20 ng/ml, Gleason score of 7, or clinical stage T2b. And high risk refers to PSA higher than 20 ng/ml, Gleason score equal or larger than 8, or clinical stage T2c-3a. A total of 241 people were categorized as low & intermediate risk and other patients (459/700) were classified as high risk. We compared the clinical factors between high risk and combination of low and intermediate risk groups (Table 2). We found that patients with a low level of HDL-c (<1.03 mmol/L) had 43.1% higher risk of high risk prostate cancer compared to men with normal HDL-c. The result of multivariate logistic regression analysis also showed that after adjusted for other preoperative clinical variables, low HDL-c level is a predictor of high risk disease (*P* = 0.042, OR: 1.469, 95%CI: 1.015- 2.126).

**Table 2:** Comparison of parameters between low & intermediate risk and high risk patients

	Low & intermediate risk	High risk	*P*	Unadjusted OR (95%CI)
	N=241(%)	N=459(%)		
Demographics				
Age at diagnosis	69.04±6.34	68.92±6.52	0.377	1.011(0.961-1.036)
Obesity (BMI≥30)	82(34.0%)	183(39.9%)	0.130	1.285(0.929-1.779)
Hypertention	80(26.1%)	168(29.4%)	0.627	0.921(0.663-1.282)
Diabetes	33(13.7%)	84(18.3%)	0.112	1.412(0.912-2.183)
Lipid profile				
HDL-c (<1.03 mmol/L)	57(23.7%)	141(30.7%)	**0.049**	**1.431(1.001-2.046)**
LDL-c(>4.1 mmol/L)	16(6.6%)	27(5.9%)	0.692	0.879(0.464-1.664)
Total cholesterol (>6.2 mmol/L)	24(10.0%)	52(11.3%)	0.580	1.155(0.693-1.927)
Total triglyceride (>2.25 mmol/L)	29(12.0%)	58(12.6%)	0.818	1.057(0.657-1.700)

Next, we divided BMI into four groups to explore the association between obesity and clinicopathological characteristics of prostate cancer (Table 3). Compared with normal weight patients, increased level of BMI is associated with higher PSA. Moderately and severely obese patients (BMI≥35 kg/m^2^) is at a higher risk of high grade of PCa (*P* = 0.007, OR: 1.961, 95%CI: 1.198-3.213) in comparison of patients with BMI < 25 kg/m^2^.

**Table 3:** Comparison of parameters between different BMI levels of patients

	Normal weight		Overweight		Mildly obese		Moderately and severely obese	
	(<25 kg/m^2^)		(25 to <30 kg/m^2^)		(30 to <35 kg/m^2^)		(≥35 kg/m^2^)	
	N=217	OR (95%CI)	N=218	OR(95%CI)	N=140	OR(95%CI)	N=125	OR(95%CI)
Age at diagnosis	70.05±6.41			69.01±6.14		67.20±6.41		67.69±6.48
*P* & OR(95%CI)	1	ref.	0.087	0.974 (0.945-1.004)	**<0.001**	**0.934 (0.902-0.967)**	**0.002**	**0.946 (0.914-0.979)**
Diagnosis of PSA(ng/ml)	20.34±19.11			98.95±535.65		110.82±452.25		96.21±443.14
*P* & OR(95%CI)	1	ref.	**<0.001**	**2.304 (1.469-3.615)**	**<0.001**	**3.144 (1.869-5.290)**	**<0.001**	**3.300 (1.852-5.880)**
Pathological stage								
T1–T2a	64		61		36		27	
T2b–T2c	99		106		69		62	
*P* & OR(95%CI)	1	ref.	0.608	1.123 (0.720-1.753)	0.411	1.239 (0.743-2.066)	0.161	1.484 (0.856-2.574)
T3+	54		51		35		36	
*P* & OR(95%CI)	1	ref.	0.972	0.991 (0.590-1.665)	0.638	1.152 (0.639-2.078)	0.146	1.580 (0.853-2.927)
Gleason score								
≤6	83		72		49		30	
≥7	134		146		91		95	
*P* & OR(95%CI)	1	ref.	0.256	1.256 (0.848-1.861)	0.535	1.150 (0.739-1.790)	**0.007**	**1.961 (1.198-3.213)**
Capsular penetration	14		19		13		13	
*P* & OR(95%CI)	1	ref.	0.374	1.384 (0.676-2.837)	0.325	1.484 (0.676-3.260)	0.196	1.683 (0.764-3.706)
Seminal vesicle invasion	22		19		17		16	
*P* & OR(95%CI)	1	ref.	0.612	0.846 (0.444-1.613)	0.554	1.225 (0.626-2.399)	0.452	1.301 (0.656-2.582)
Lymph node involvement	25		29		21		9	
*P* & OR(95%CI)	1	ref.	0.573	1.178 (0.666-2.087)	0.339	1.355 (0.726-2.528)	0.202	0.596 (0.269-1.321)

**Figure 1:**
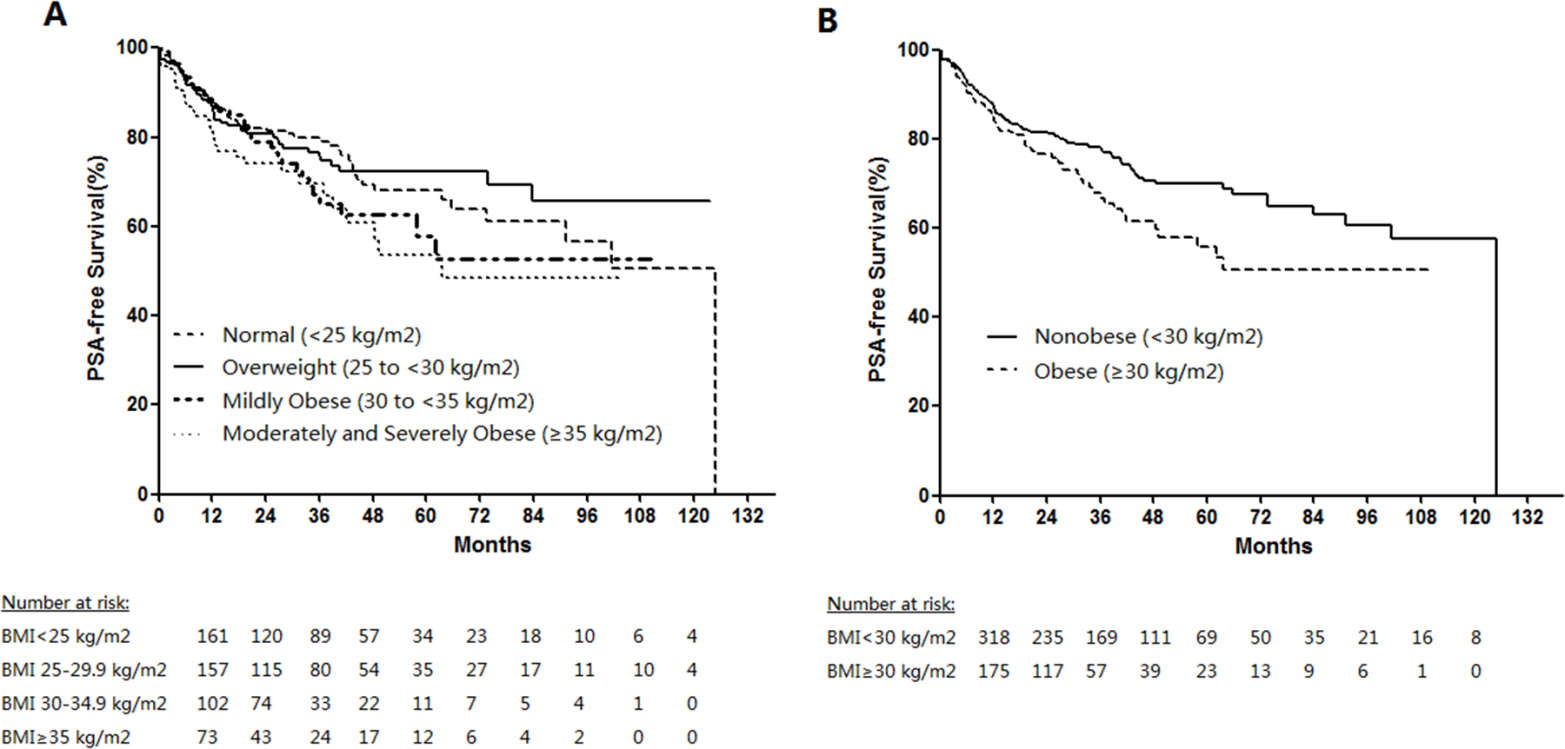
PSA-free survival percentage estimates by body mass index (BMI) after thirteen-years follow-up Log-rank *P* values. **(A)** Normal vs. overweight, *P* = 0.766; normal vs. mildly obese, *P* = 0.255; normal vs. moderately and severely obese, *P* = 0.045; overweight vs. mildly obese, *P* = 0.262; overweight vs. moderately and severely obese, *P* = 0.042; mildly obese vs. moderately and severely obese, *P* = 0.361. **(B)** Nonobese vs. obese, *P* = 0.027.

In addition, we performed survival analysis to further investigate the influence of obesity on disease progress of PCa. Of the 700 PCa patients, the median follow-up after RP was 85 months (interquartile range 4 – 123 months). 175 patients (25.0%) developed a biochemical recurrence. Kaplan-Meier method was used to compare the PSA-free survival percentage between the different groups of BMI. As is shown in Figure 1A, patients with normal weight (BMI < 25 kg/m^2^) were significantly less likely to develop a biochemical recurrence compared with the moderately and severely obese patients (*P* = 0.045). When we performed combined analysis, obese patients (BMI ≥ 30 kg/m^2^) had significantly higher rates of PSA recurrence over time than nonobese (BMI < 30 kg/m^2^) patients (*P* = 0.027; Figure 1B). Univariate and multivariate COX analysis were conducted to select the predictive factors of disease progress. On univariate analysis, higher BMI (*P* = 0.028, HR: 1.405, 95%CI: 1.405-1.903) and the presence of common disease characteristics like higher PSA level (*P* < 0.001, HR: 2.647, 95%CI: 2.068-3.389), Gleason Score (*P* < 0.001, HR:2.359, 95%CI: 1.661-3.351), pathologic T-stage (*P* < 0.001, HR: 5.799, 95%CI: 3.292-10.215), seminal vesicle invasion (*P* = 0.001, HR:1.996, 95%CI: 1.321-3.016) and positive lymph node involvement (*P* < 0.001, HR: 2.926, 95%CI: 1.995-4.290) were associated with increasing rate of PSA recurrence. However on multivariate analysis, BMI and seminal vesicle invasion didn’t remain to be independent predictors of cancer recurrence (Table 4).

**Table 4:** Cox proportional hazards analysis of factors predicting time to biochemical (PSA) recurrence after Radical prostatectomy

Factors	Hazard ratio	95% CI	*P*
Univariate analysis			
BMI(obese vs. nonobese)	1.405	1.405-1.903	0.028
Log PSA(continuous)	2.647	2.068-3.389	<0.001
Gleason score(≥7 vs. ≤6)	2.359	1.661-3.351	<0.001
Pathologic T-stage(≥T2b vs. ≤T2a)	5.799	3.292-10.215	<0.001
Seminal vesicle invasion(Yes vs. No)	1.996	1.321-3.016	0.001
Lymph node involvement(Yes vs. No)	2.926	1.995-4.290	<0.001
Multivariate analysis			
Gleason score(≤6 vs. ≥7)	1.497	1.029-2.180	0.035
Pathologic T-stage	4.698	2.589-8.523	<0.001
Lymph node involvement	1.630	1.087-2.445	0.018
Log PSA(continuous)	1.952	1.477-2.579	<0.001

## DISCUSSION

The association between obesity and the risk of prostate cancer has long been widely investigated, but the results across the studies remain inconsistent [6–11]. In the present study, we observed no association between BMI and the risk of prostate cancer, which differed from previous familiar studies including Chinese population [8]. Lipid profile, including HDL-c, LDL-c, total triglyceride and total cholesterol, are modifiable factors which can reflect the condition of obesity. Our study suggests that the dyslipidemia may be associated with a higher possibility of prostate cancer, including a low level of HDL-c and high level of LDL-c and total cholesterol, which is similar with the study in Asia [28]. Hypertriglyceridemia has also weakly positive association with PCa, although it hasn’t reached the statistical significance. According to the studies in Indian and European population, dyslipedimia could be related to increased tumor grade [24, 29]. Our study also found that aberrant HDL-c level is a strong predictor of developing high risk disease. As statins may not only lower total cholesterol but also tend to raise HDL-c, previous studies reported that the risk of aggressive or high-grade prostate cancer reduced among men who use statin drugs [30–33] , which make our findings credible.

We further stratified the analysis based on BMI to investigate the influence of the obesity on the outcome of PCa. PSA at diagnosis is significantly higher in other categories of BMI when compared with the normal categories. In addition, the risk of PCa with Gleason Score ≥ 7 increased 96.1% in moderately and severely categories, indicating that obesity may be a significant risk factor of the outcome of PCa patients receive RP. Univariate Cox proportional hazards analysis was conducted to confirm our hypothesis. We find that increased BMI also predicted higher biochemical recurrence rates, just like the adverse pathologic variables such as PSA, capsular penetration, lymph node involvement and seminal vesicle invasion. The similar results can be found in the Center for Prostate Disease Research in USA [14].

Although the association between obesity with dyslipedimia and PCa has been widely studied, the mechanism underlying is not clearly understood. Many hypotheses have been proposed to explain inter-relationship between lipid and PCa development and progression. It is commonly recognized that epithelial cells of normal prostate have higher cholesterol content compared with other normal cells of organs across the whole body. During the progression of prostate cancer, the cholesterol content gets even higher [34], indicating that the accumulation of cholesterol may contribute to the malignant conversion of prostate. Specifically, the over-accumulate cholesterol forms large lipid rafts in the cell membranes of prostate cancer cells, which may promote the cell signing in carcinogenesis [35]. Pathways such as Akt pathways and the sonic hedgehog have been proved to be cholesterol sensitive [36, 37] and facilitate the growth of PCa cell lines both *in vitro* and in xenograft models [38]. Since the role of cholesterol in PCa progression, low level of cholesterol may suppress the pro-carcinogenic activities. HDL-c cholesterol, which can transport cholesterol from cells to the liver and other steroidogenic organs [39], is thought to be a powerful factor to remove harmful cholesterol from prostate and lower the risk of PCa through the mechanisms discussed above. Additionally, it is reported that HDL-c have functions of anti-inflammatory and antioxidant, which may reduce growth and progression activity of prostate cancer [40]. High level of triglycerides has also been demonstrated to be responsible for PCa development by elevating levels of reactive oxygen species and oxidative stress and developing insulin resistance [41].

It has been known to us that adipose tissue can not only play a role of energy storage, but also act as endocrine organ [42]. Multiple hormones secreted by the fat tissue can activate alternative pathways that effect physiological function of the body system or promote pathological procedure. The increased levels of serum estradiol in men with obesity lead to an inhibition feedback of pituitary- hypothalamic axis, resulting in low levels of serum free testosterone [43]. Some studies have demonstrated that decreased serum testosterone levels were associated with high grade and advanced tumors [44, 45]. And low testosterone level predicts increased risk for disease upgrading and upstaging in active surveillance (AS) prostate cancer patients who elected to undergo radical prostatectomy [46]. One possible explanation is that testosterone promotes the differentiation prostate epithelium cells, and low level of testosterone activity may promote PCa cells differentiation, causing differentiated tumors. Obesity is also known associated with high serum insulin and insulin resistance, which are risk factors of developed PCa. Along with hyperinsulinemima, insulin growth factors (IGFs) elevation is also observed in obese men [47–49]. PCa cell lines cultured with the patients serum obtained the changes above had significantly high mitogenic activity *in vitro* [50, 51], leading to larger, advanced and metastatic diseases. IGFBP-3, which is the binding protein of IGF in serum, exerts its biological role by binding and sequestering IGF-1 molecules to suppress cell growth under physiologic circumstances. It has been showed to be inversely associated with Gleason score [52] and can be an important predictor for Gleason Score migration [53]. Furthermore, increased serum fatty acid levels and leptin are also proved to be capable of predicting poor prognosis of PCa patients with obesity [54–59]. In addition to the biologic processes discussed above, obesity is always linked with technical difficulties in radical prostatectomy, which is often referred with larger prostate volume and bad surgery vision, causing an increased risk of positive surgical margins. That is another reason of higher recurrence rates among obese men [13, 14, 60].

In conclusion, we are the first to demonstrate the association between the lipid profile and the risk of prostate cancer and further explore the predictors of PSA recurrence in the largest cohort of Chinese population. In our study, low level of HDL-c and high level of LDL-c and total cholesterol are supposed to contribute to the pathogenesis of PCa. Our data also support the hypothesis that obese men are at increased risk of PSA recurrence after RP. With the development of China, obesity and prostate cancer have been increasingly prevalent among Chinese men. We hope our findings could arouse the concern of people on this health problem.

## MATERIALS AND METHODS

### Study population

This study was performed at the Departments of Urology in the First Affiliated Hospital of Nanjing Medical University between Dec 2004 and Feb 2014. Parameters like age, PSA, digital rectal examination (DRE) findings, height, weight, lipid profile and other clinical information of patients with complains of urological system who visited our departments were recorded in detail. Patients with serum PSA greater than 4 ng/ml or/and abnormal DRE or/and hypoecho under the transrectal ultrasound (TRUS) were requested to receive (TRUS)-guided prostate biopsy. The exclusion criteria were (i) patients having any chronic disease like HIV/AIDS, tuberculosis, or major endocrinopathies such as thyroid and adrenal dysfunction, (ii) patients with history of other cancers or relative therapy before, (iii) repeat biopsy and (iv) records with incomplete data. At last, a total of 974 consecutive men were diagnosed as PCa and 700 patients subsequently received radical prostatectomy (RP) immediately, and 1031 people were diagnosed as biopsy negative (refer to non-PCa group in the following text) after screen records of 3102 patients. All patients who received RP were asked to have regular follow-ups including PSA and lipid profile every 3-6 months.

Prostate biopsy and RP were performed by the same one skilled surgical doctor and his team. Sample tissues of biopsy or RP were sent to pathology department for histological examination and upon histological confirmation of prostate cancer or non-PCa people. BMI (kg/m^2^) was calculated as weight in kilograms divided by height in meters squared and classify men into four categories using the National Institutes of Health definitions of normal weight (<25.0 kg/m^2^), overweight (≥25.0 to <30.0 kg/m^2^), mildly obese (≥30.0 to <35.0 kg/m^2^) and moderately and severely obese (≥35.0 kg/m^2^). Blood sample of the patients were collected after more than 12 hours of fast in the morning and no drinking and no smoking were advised. Hypertension is defined as systolic pressure >140 mmHg or/and diastolic pressure >90mmHg. Biopsy specimens containing adenocarcinoma were scored according to the Gleason grading system. Biochemical recurrence was defined as two consecutive tests of PSA>0.2 ng/ml in two weeks.

This study was approved by the institutional review board of the First Affiliated Hospital of Nanjing Medical University. Written informed consent was obtained from all patients with regard to the storage of their information for the purpose of research. All research procedures were conducted in accordance with the Declaration of Helsinki.

### Statistics

All analyses were performed using SPSS 18.0. Kruskal-Wallis test for continuous variables and the chi-squared tests for categorical variables were used for univariate comparison of the differences in patient characteristics across BMI categories between different groups. Odds ratios (OR) and 95% confidence intervals (CI) were estimated for the association between PCa and the various patient characteristics. Multivariable Cox proportional hazards regression was performed to assess the risk of PCa recurrence. *P*-values were 2-sided, and *P*-values < 0.05 were chosen for significance.

## Abbreviations

(PCa): Prostate cancer
(BMI): body mass index
(PSA): prostate-specific antigen
(RP): radical prostatectomy
(OR): odds ratios
(95% CI): 95% confidence intervals
(LDL-c): low-density lipoprotein cholesterol
(HDL-c): high-density lipoprotein cholesterol
(DRE): digital rectal examination
